# Mohs Defect Repair with Dehydrated Human Amnion/Chorion Membrane

**DOI:** 10.1089/fpsam.2021.0167

**Published:** 2022-01-03

**Authors:** Julia Toman, Georgina M. Michael, Oliver J. Wisco, John R. Adams, Brandon S. Hubbs

**Affiliations:** ^1^ Division of Facial Plastics and Reconstructive Surgery, Department of Otolaryngology Head and Neck Surgery, University of South Florida, Tampa, Florida, USA.; ^2^ Department of Clinical Research, MiMedx Group, Inc., Marietta, Georgia, USA.; ^3^ Dermatology Health Specialists, Bend, Oregon, USA.; ^4^ Department of Dermatology, The Warren Alpert Medical School of Brown University, Providence, Rhode Island, USA.; ^5^ Advanced Dermatology and Skin Cancer Center, Manhattan, Kansas, USA.; ^6^ Division of Dermatology, Department of Internal Medicine, University of Kansas School of Medicine, Wichita, Kansas, USA.; ^7^ Biostatistics Consulting, Alpharetta, GA, USA.

## Abstract

**Importance:** Reconstructing cosmetically sensitive defects in an aging population undergoing multiple Mohs micrographic surgeries (MMS) may be addressed with alternatives to surgery.

**Objective:** Patients undergoing MMS with defect reconstruction in visually prominent areas receiving placental allograft were compared with traditional autologous tissue-based procedures—flaps and full-thickness skin grafts (FTSG).

**Design, Setting, and Participants:** This retrospective case–control study evaluated patients who underwent MMS for removal of a basal or squamous cell carcinoma with same-day repair.

**Main Outcomes and Measures:** The primary endpoint was the incidence and comparison of postoperative morbidity. Risk for developing medical or cosmetic sequelae was determined through multivariate logistic regression.

**Results:** The study population consisted of 143 propensity score-matched pairs (*n* = 286) with moderate- to high-risk defects on the face, head, and neck. Compared with autologous tissue, placental allograft cases were associated with significantly lower risk for infection (*p* = 0.004), poor scar cosmesis (*p* < 0.0001), scar revision (*p* < 0.0001), or reoperation (*p* = 0.0007).

**Conclusions and Relevance:** Postoperative complication rates for placental reconstructions did not exceed those demonstrated by autologous tissue counterparts, indicating this is a safe alternative to flap and FTSG in cosmetically sensitive repairs.

**Key Points**

**Question:** Is the use of a placental allograft a feasible alternative to incisional methods of repair for cosmetically sensitive defects in select patient cases after Mohs surgery?

**Findings:** Larger cutaneous Mohs-related defects of the face, head, and hands were effectively reconstructed with a placental allograft in a population of older adults.

**Meaning:** This study suggests that surgical reconstruction after skin cancer removal from the face may be avoided in some cases when treated with a placental-derived material.

## Introduction

Epidemiological estimates indicate a disproportionate surge of nonmelanoma skin cancer (NMSC) in the elderly, with 80% of all new cases occurring in persons >65 years.^1,2^ As a result of longer life expectancy and the cumulative impact of ultraviolet radiation and sun exposure on anatomically vulnerable sites such as the face, head, and neck, older individuals may develop multiple tumors in localized regions.^3,4^ This necessitates repeat surgical management that maintains a delicate balance between adequate excision, aesthetics, and function.^5–7^

Incisional repair utilizing autologous tissue such as local flaps and full-thickness skin grafts (FTSG) represent the mainstay of reconstructive techniques after Mohs micrographic surgery (MMS).^8,9^ However, postoperative morbidity increases after the age of 60 years by as much as 25% due to a greater depth of tumor invasion and extensive defects with higher demands for donor tissues.^10,11^ Age and previous surgery alter characteristics of surrounding tissues and complicate the aesthetic and functional success of flaps and FTSG.^12,13^ Medically fragile patients or those who are ineligible for incisional repair may be limited to second intention healing, a method typically reserved for smaller concave surfaces.^14^

Larger MMS defects that do not receive definitive reconstruction have been associated with increased recovery time, delayed healing, wound retraction, unpredictable scarring, and poor cosmesis.^15^ This can lead to an altered appearance or impairment, causing patients to feel a sense of disfigurement, emotional distress, social isolation, or diminished quality of life.^16–18^

In situations where incisional repair options are limited, an ideal alternative would be a nonsurgical approach capable of yielding outcomes comparable with autologous tissue techniques. Placental tissues may offer a novel solution to address this emerging clinical need in cutaneous reconstruction. These commercially available allografts retain the three-dimensional collagen-rich (types I, III, IV, V, and VII) extracellular matrix, growth factors (bFGF, basic fibroblast growth factor; VEGF, vascular endothelial growth factor; PDGF, platelet-derived growth factor; EGF, epidermal growth factor; SDF-1, stromal cell-derived factor-1; and TGFβ-3, transforming growth factor beta-3), cytokines (IL-10, interleukin 10; IL-1Ra, interleukin-1 receptor antagonist; and TIMPs, tissue inhibitors of metalloproteinases), antimicrobial peptides (NGAL, neutrophil gelatinase associated lipocalin; LL-37, human cationic antibacterial protein of 18 kDA; and RNase7, ribonuclease 7), and endogenous cells—epithelial cells, fibroblasts, and mesenchymal stem cells—native to human placental tissues.^19,20^ Nearly all Level 1 randomized clinical trial placental allograft data exist in the chronic wound care space.^21^ Low immunogenicity, ease of transplantation, and anti-inflammatory, angiogenic^*^, and antiscarring properties suggest that this biomaterial is particularly well suited for a variety of clinical applications.^22–24^

To date, no controlled study has investigated placental allografts in the reconstructive management of moderate- and high-risk MMS defects in cosmetically sensitive areas. The primary purpose of this analysis is to assess the safety and utility of a dehydrated human amnion/chorion membrane (dHACM) (EpiFix^®^; MiMedx Group Inc., Marietta, GA) as a nonsurgical approach to cutaneous reconstruction. When compared in equivalent cases, we hypothesized that postoperative outcomes would be statistically similar to those achieved with flap and FTSG.

## Methods

### Study design

To ensure accurate and transparent reporting, this retrospective case–control study was conducted according to the Strengthening the Reporting of Observational Studies in Epidemiology guidelines.^25^ Institutional Review Board approval and waiver of informed consent (Pro0031033) was granted before review of electronic medical records and extraction of deidentified data between January 2014 and December 2018. Patients were included if their diagnosis was basal or squamous cell carcinoma, and if the MMS defect was located in a moderate- to high-risk area (face, head, neck, or dorsal hand), and necessitated same-day reconstruction. The American Academy of Dermatology Mohs Appropriate Use Criteria was used to assign reconstructive complexity and stratify defects according to location and aesthetic subunit.^26^

After exclusions, a total of 1550 eligible patients were identified. Cases were categorized based on the modality of same-day reconstruction. In this study, reconstructions were dichotomized into two groups, defined as either autologous tissue (*n* = 1397)—flaps and FTSG—or placental allograft (dHACM) (*n* = 153). To avoid selection bias and ensure a balanced comparison, propensity scores were constructed for each patient. Cases without an equivalent match were discarded. The final study population consisted of 286 patients that could be propensity score-matched at a 1:1 ratio, generating 143 case–control pairs.

### Outcome measures

**Fig. 1.**
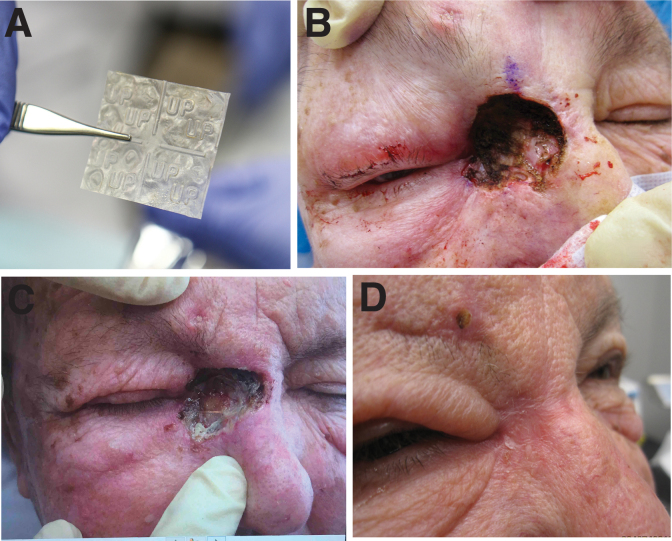
**(A)** Placental allograft (dHACM) out of package before implantation (additional detail regarding product application is provided in the manufacturer's instructions for use [IFU], provided as Supplemental Data). **(B)** Mohs patient with large cutaneous defect involving upper eyelid and medial canthus right eye and right upper bridge of nose. **(C)** Placental allograft in situ, remains visible in situ at postoperative day 7. **(D)** Complete closure of Mohs defect without complication to surrounding structures, postoperative day 42. dHACM, dehydrated human amnion/chorion membrane.

All MMS and reconstructions were managed with the same postoperative protocol. Prophylactic antibiotics were not prescribed. Patients were evaluated every 5–7 days and received appropriate site care and dressing changes. Placental allograft applications were repeated if the previous graft material was no longer visible in the wound bed (Fig. 1A–C). Subjects were discharged from care once reconstruction sites were healed and free from complication (Fig. 1D).

The primary endpoint was the incidence of postoperative morbidity. Comparisons included the rate of medical complications for the index site: (1) infection, (2) bleeding/hematoma, (3) dehiscence, (4) surgical reintervention, or (5) development of a nonhealing wound. Postoperative cosmetic outcomes were assessed at ≥9 months. These measures included documentation of (1) suboptimal scarring (per the International Classification of Diseases, Tenth Revision, Clinical Modification; ICD-10-CM-L90.5 cicatrix descriptors: adherent, painful, hypertrophic, contracted, fibrotic, or ectropion), (2) scar revision/treatment (excision, debulking, intralesional injection, laser, and dermabrasion), and (3) patient satisfaction with scar appearance. The length of time (days) and total number of visits required before discharge (determined by complete wound closure), and the billed costs associated with the MMS and primary reconstruction were also evaluated.

### Statistical analysis

The statistical software program SAS/STAT^®^ v9.4 (SAS Institute Inc., Cary, NC) analyzed deidentified study data. Descriptive statistics for continuous variables were expressed as means and standard deviations (SDs). Frequencies (*n*) and percentages summarized dichotomous variables and categorical observations. Differences were compared with Student's *t*-test, Fisher's exact test, or Pearson's χ^2^ test. Use of nonparametric Mann–Whitney test or Wilcoxon rank-sum methods depended on the distribution of data. All statistical tests were two-sided and results were regarded as statistically significant at *p* < 0.05.

Propensity scores for matched case–controls were estimated using a logistic regression model to correct for differences in covariates: gender, age in years, medical history, tumor size, tumor location, histopathology, and other MMS characteristics. The goodness of propensity score-matched pairs was evaluated using signed-rank sum for continuous data and McNemar's test for binary data. Adjusted multivariate odds ratio (OR; 95% confidence interval [CI]) determined predictors of significant postoperative morbidity and relative risk ratios (RR; 95% CI) were used to assess the strength of associations in univariate comparisons.

## Results

Baseline characteristics for unmatched and matched patients are summarized in Table 1. Significant covariate imbalances were corrected in the matched case–control sample (*n* = 286). Males represented the majority gender in both groups (*p* = 0.161). The mean (SD) ages ranged from 78.0 to 78.8 years (*p* = 0.454). There were no significant differences in medical history, tumor size, tumor type, moderate- versus high-risk defect location, or operative time (*p* > 0.05).

**Table 1.** Patient, tumor, and Mohs micrographic surgery characteristics for entire sample and matched sample

	Entire sample			Matched sample		
	Placental allograft (*n* = 153)	Autologous flap/FTSG (*n* = 1397)	p	Placental allograft (*n* = 143)	Autologous flap/FTSG (*n* = 143)	p
White	153 (100.0)	1397 (100.0)	—	143 (100.0)	143 (100.0)	—
Male, *n*	122 (79.7)	1038 (74.3)	0.141	115 (80.4)	105 (73.4)	0.160
Age, years	78.4 (9.5)	76.0 (9.4)	0.003	78.0 (9.6)	78.8 (9.1)	0.454
Immunocompromised, *n*	33 (21.6)	286 (20.5)	0.750	32 (22.4)	23 (16.1)	0.177
Anticoagulation, *n*	36 (23.5)	344 (24.6)	0.765	34 (23.8)	39 (27.3)	0.497
Current smoker, *n*	16 (10.5)	131 (9.4)	0.868	16 (11.2)	17 (11.9)	0.600
Tumor type (NMSC), *n*			0.408			0.233
Basal cell	38 (24.8)	392 (28.1)		35 (24.5)	39 (27.3)	
Squamous cell	115 (75.2)	996 (71.3)		108 (75.5)	104 (72.7)	
Other	0 (0.0)	9 (0.6)		0 (0.0)	0 (0.0)	
Tumor length, cm	1.3 (0.7)	1.4 (0.7)	0.027	1.3 (7.0)	1.3 (6.0)	0.692
Tumor width, cm	1.0 (0.4)	1.1 (0.5)	<0.001	1.0 (4.0)	1.0 (3.0)	0.929
MMS AUC area			0.213			0.233
Area M	33 (21.6)	366 (26.2)		32 (22.4)	24 (16.8)	
Area H	120 (78.4)	1031 (73.8)		111 (77.6)	119 (83.2)	
MMS time, minutes	195.8 (62.4)	237.6 (50.1)	<0.001	197.4 (57.5)	199.9 (44.4)	0.677

Data are expressed as absolute number (*n*) and percent (%) or mean and standard deviation (SD); statistical significance at *p* < 0.05.

Allograft, placental (dHACM, dehydrated human amnion/chorion membrane); Autologous tissue, flap and full-thickness skin graft (FTSG); NMSC, non-melanoma skin cancers; AUC, Appropriate Use Criteria per American Academy of Dermatology; Area H (high risk), central face, eyelids [including inner/outer canthi], eyebrows, nose, lips [including cutaneous surround, mucosal area, vermillion border], chin, ear [including periauricular skin/sulci], temple, and hands; Area M (moderate risk), cheeks, forehead, scalp, neck, and jawline; MMS time, length of Mohs procedure in minutes; NMSC, non-melanoma skin cancers.

### Univariate analysis

The univariate comparison of outcomes is summarized in Table 2. The mean (SD) size of MMS defects reconstructed with placental allograft were similar to autologous tissue, 3.5 (3.7) versus 3.3 (3.1) cm^2^, respectively (*p* = 0.531). A significantly greater proportion of placental allograft patients (97.9%) experienced zero postoperative complications compared with autologous tissue (71.3%), *p* < 0.0001^*^ (Supplementary Figs. 1–3); strength of association was confirmed by relative risk measures (RR = 13.67; 95% CI = 4.33–43.12).

**Table 2.** Univariate analysis for outcomes in case–control comparisons

	Placental allograft (*n* = 143)	Autologous flap/FTSG (*n* = 143)	p
Size of MMS defect, cm^2^	3.5 (3.7)	3.3 (3.1)	0.531
Stages for tumor clearance	2.0 (1.2)	2.1 (1.1)	0.605
Experienced no complications	140.0 (97.9)	102.0 (71.3)	<0.0001
Postoperative sequelae			
Infection	3.0 (2.0)	15.0 (10.0)	0.004
Bleeding or hematoma	0.0 (0.0)	7.0 (5.0)	0.015
Wound dehiscence	0.0 (0.0)	4.0 (3.0)	0.122
Surgical reintervention	0.0 (0.0)	11.0 (8.0)	0.0007
Nonhealing wound	0.0 (0.0)	5.0 (3.5)	0.060
Poor scar cosmesis	0.0 (0.0)	21.0 (15.0)	<0.0001
Scar revision	0.0 (0.0)	14.0 (9.8)	<0.0001
Follow-up visits	3.4 (1.6)	2.5 (1.1)	<0.0001
Time to discharge, days	30.7 (16.9)	30.3 (22.9)	0.840
Cost of reconstruction, dollars	4463 (2272)	3904 (951)	0.007

Data are expressed as absolute number (*n*) and percent (%) or mean and standard deviation (SD); statistical significance at *p* < 0.05.

Placental allograft reconstructions developed less infection (*p* = 0.004) and were less likely to experience poor scar cosmesis (*p* < 0.0001) (Supplementary Figs. 4 and 5), scar revision (*p* < 0.0001), or surgical reintervention at the index site (*p* = 0.0007). Autologous tissue reconstructions required fewer mean (SD) follow-up visits (2.5 [1.1] versus 3.4 [1.6] visits; *p* < 0.0001) and cost less (3,904 [951] versus 4,463 [2,272] dollars; *p* = 0.007). The number of days to discharge were not significantly different between groups 30.7 (16.9) versus 30.3 (22.9) days (*p* = 0.840).

### Multivariate analysis

Logistic regression model results are depicted in Table 3. When controlling for defect surface area, operation time, age, medical history, and gender, autologous tissue reconstruction remained an independent significant risk factor for infection or additional operation (OR = 11.71; 95% CI = 3.35–40.99; *p* < 0.0001). In a separate model that included cosmetic outcomes, the odds of infection, additional operation, poor scar cosmesis, or scar revision were 19 times higher in the autologous tissue group (OR = 18.76; 95% CI = 5.56–63.34; *p* < 0.0001). Being female was also associated with three times greater odds of having a cosmetic complication (OR = 2.84; 95% CI = 1.29–6.23; *p* = 0.010).

**Table 3.** Multivariate logistic regression analysis for the risk of medical and cosmetic postoperative complications

	Infection or surgical reintervention		Poor scar cosmesis or scar revision	
	OR (95% CI)	p	OR (95% CI)	p
Female gender	1.68 (0.68–4.15)	0.260	2.84 (1.29–6.23)	0.009
Age	0.98 (0.94–1.03)	0.481	1.00 (0.96–1.05)	0.861
Tumor size, cm^2^	1.27 (0.82–1.97)	0.279	1.06 (0.74–1.52)	0.755
Defect size, cm^2^	0.91 (0.71–1.17)	0.470	0.97 (0.82–1.14)	0.683
Central face defect location	0.45 (0.17–1.24)	0.123	0.77 (0.30–2.01)	0.593
Autologous tissue vs. placental allograft	11.71 (3.35–40.99)	0.0001	18.76 (5.56–63.34)	<0.0001
Current smoker	0.74 (0.17–3.14)	0.677	0.92 (0.27–3.08)	0.886
Anticoagulation	1.00 (0.39–2.61)	0.997	0.98 (0.43–2.23)	0.953
Immunocompromised	1.32 (0.45–3.85)	0.618	0.92 (0.33–2.56)	0.873
No. stages to extirpation	1.04 (0.71–1.53)	0.846	1.16 (0.84–1.61)	0.363
Length of surgery	1.00 (1.00–1.01)	0.447	1.00 (1.00–1.01)	0.419

Statistical significance at *p* < 0.05.

CI, confidence interval; OR, odds ratio.

## Discussion

This propensity score-matched case–control is the first to compare the outcomes for placental allograft with the gold standard of management in complex MMS defect reconstruction, flaps, and FTSG. This study presented a unique opportunity to address a practice gap with evidence that is currently missing from the literature. The key findings of this investigation are the incidence of infection and all-cause postoperative morbidity in placental allograft repairs was significantly lower than observed with autologous tissue; and the use of placental allograft was independently associated with a lower risk of infection, repeat operation, poor scar cosmesis, and scar revision. Even after controlling for covariates, patients receiving autologous tissue reconstruction were 12 times more likely to have infections or surgical reintervention and 19 times more likely to experience poor scar cosmesis or scar revision. Although female patients were at a greater risk of having postoperative scar-related issues, such outcomes coincide^*^ with previous studies in which females experienced more psychosocial distress and scar concerns after MMS in a visually prominent area.^18^

Our study findings are consistent with long-standing reports in the scientific and medical literature, which recognize the placental membrane as a safe and effective surgical material.^19–24^ Considerable research supports its use for tissue engineering as a biocompatible scaffold with capacity to promote re-epithelialization while reducing wound fibrosis and scar formation.^20^ The successful closure of chronic wounds, management of orthopedic injury, prevention of surgical tissue adhesions, ocular surface reconstruction, reduced pain and infection in burn treatment, and prevention of postoperative hematoma and serous fluid collections are evidenced through various levels of research.^21,22^

In the dermatologic surgery setting, placental allografts may optimize clinical results in a variety of circumstances where a combination of patient, anatomical, and tissue-related factors are a concern: individual refuses incisional repair or presents with a medical contraindication; the quality/availability of local donor tissues are challenged; underlying structures are exposed and require a protective barrier; free skin margin contraction may lead to impaired cosmesis/altered function; or, the area cannot be immobilized to maintain autograft survival.^23^

The treatment algorithm for placental allograft use would supplement, not substitute for, levels in the reconstructive ladder. In this study, defects managed with placental allograft were characterized by exposed muscle, bone, and cartilage. These large complex wounds required one additional visit and cost an average $560 more than autologous tissue techniques. This can be attributed to repeat application of the placental tissue and cost of the graft. Despite increased health care utilization concerns, this nonsurgical method became an option—when due to a combination of patient, anatomical, and tissue-related factors—the optimal reconstruction method could not be pursued.

### Limitations

As with all observational data, these study findings must be interpreted in the context of the retrospective study design. Results are limited by the accuracy and thoroughness of medical records. Given the resources available, this investigation made extensive efforts to increase internal validity and limit the confounding of results. Although propensity score-matching reduced baseline covariate imbalances, it does not substitute for randomization. A demographically homogenous cohort also constrains the generalizability of findings. The inherent risks associated with each reconstructive modality, incisional repair versus allograft tissue transplantation, present another limitation. The aim of this study was to compare defects distinguished by equivalent reconstructive demands in terms of complexity, location, and size. Smaller MMS defects managed with second intention were distinctly different (shallow depth, concave surfaces, and low-risk location) and, therefore, a case–control match with a population of dissimilar wounds was not applicable.

## Conclusion

Placental allograft can be a safe and effective tool for repairing MMS defects of the face, head, neck, and dorsal hand in a subset of patients who are not good candidates for traditional methods of autologous tissue reconstruction. In the authors' experience, this includes older adults with moderate- to high-risk defects >3 cm^2^ and medically comorbid individuals with wound bed concerns making them ineligible for incisional repair. Further investigation into the efficacy of placental allograft for MMS reconstruction among racially diverse groups needs to be addressed in future studies.

## Supplementary Material

Supplemental data

Supplemental data

Supplemental data

Supplemental data

## Notes

^
*****
^
**Correction added** on November 8, 2021 after first online publication of October 29, 2021: In the sentence, “A significantly greater proportion…” the *p* value was mistakenly added as *p* ≤ 0.0001. This value is now corrected as *p* < 0.0001.

^
*****
^
**Correction added on** November 8, 2021 after first online publication of October 29, 2021: In the sentence, “Low immunogenicity, ease of transplantation…” the word “antiangiogenic” has been revised to “angiogenic”.

^*^
**Correction added** on November 8, 2021 after first online publication of October 29, 2021: In the sentence, “Although female patients…” the word “consistent” was mistakenly added. This has been corrected, so that the sentence reads as, “Although female patients were at a greater risk of having postoperative scar-related issues, such outcomes coincide with previous studies in which females experienced more psychosocial distress and scar concerns after MMS in a visually prominent area.”
