# Supplementary material for: Mohs Defect Repair with Dehydrated Human Amnion/Chorion Membrane
Source: Facial Plast Surg Aesthet Med. 2022 Jan 3;24(1):48–53. doi: 10.1089/fpsam.2021.0167 (PMC8783622; doi:10.1089/fpsam.2021.0167)
Supplement: Supplemental data [file Suppl_Data.pdf]

EpiFix®

Dehydrated Human Amnion/Chorion
Membrane Allograft
Information/Instructions for Use

MiMedx

1775 West Oak Commons Court NE
Marietta, GA 30062

866.477.4219

www.mimedx.com

ES101.004

EpiFix® Description

Human amniotic membrane is a thin, collagenous membrane derived from the placenta, the area in which the human fetus grows and develops within the mother's uterus. Human amniotic membrane consists of multiple layers.

EpiFix is a minimally manipulated, dehydrated, non-viable cellular amniotic membrane allograft that contains multiple extracellular matrix proteins, growth factors, cytokines and other specialty proteins present in amniotic tissue to provide a barrier membrane that enhances healing.

EpiFix allografts are human tissue products and appearance may vary between donors. Variations in color (tan to light brown), opacity, and thickness are normal due to the nature of human tissue.

Tissue Uses

EpiFix Amniotic Membrane Allograft is intended for homologous use in the treatment of acute and chronic wounds to reduce scar tissue formation, modulate inflammation, provide a barrier, and enhance healing.

Contraindications

EpiFix should not be used on (1) areas with active or latent infection and/or (2) a patient with a disorder that would create an unacceptable risk of post-operative complications.

Precautions/Warnings

- EpiFix allografts remain suitable for transplantation in an unopened, undamaged package, under proper storage conditions.
- Please inspect the integrity of the package upon receipt. If package and contents appear defective or damaged in any way, immediately contact the distributor.
- This allograft is intended for single-patient use only. Discard all unused material.
- The procedure should be performed by an authorized medical professional.
- Strict donor screening and laboratory testing, along with dedicated processing and sterilization methods, are employed to reduce the risk of any disease transmission. However, as with all biological implants, an absolute guarantee of tissue safety is not possible. This allograft has the potential to transmit infectious disease to the recipient.
- The reaction of the body to any biological implant is not completely understood.
- Caution should be used when treating patients with a known sensitivity to aminoglycoside antibiotics.
- Discard all damaged, mishandled or potentially contaminated tissue.
- This product has not been tested in combination with other products.
- DO NOT RE-STERILIZE.

Preparation, Reconstitution and Use

Prior to implantation, carefully follow the EpiFix allograft preparation steps below using aseptic technique:

Wound Bed Preparation

- Ensure the wound is free from clinical signs of infection.
- Prepare wound bed as needed.

Removing EpiFix from Packaging

- The outer peel pouch is NOT sterile. The inner pouch that contains EpiFix is sterile (unless the pouches are damaged or compromised).
- Carefully open the peelable corner of the outer pouch and extract the inner pouch using aseptic technique. Ensure the inner pouch does not come in contact with any portions of non-sterile surface of the outer pouch.
- Using aseptic technique, SLOWLY peel a corner of the inner peel pouch and grasp EpiFix with fingers or non-toothed, sterile forceps.
- Use EpiFix promptly after opening the inner, sterile pouch.

PLEASE TAKE GREAT CARE WHEN REMOVING EpiFix FROM THE INTERNAL POUCH. EpiFix IS THIN AND EXTREMELY LIGHTWEIGHT.

EpiFix Preparation

1. In a dry state, use sterile dry scissors to cut EpiFix to fit within the wound margins. It is acceptable to overlap the wound margins with EpiFix by 1mm.
2. EpiFix can be applied wet or dry.
3. EpiFix can be hydrated while on the wound site with sterile saline solution. Simply apply several drops of sterile solution to EpiFix. During and following hydration, the embossment on EpiFix will begin to fade.

EpiFix Orientation & Application

EpiFix should be placed on the wound site, using the orientation of the embossment lettering as a guide. Proper orientation of EpiFix can be noted when the embossment nomenclature reads correctly from left to right. If the mesh process has diminished the ability to clearly read the embossment, the following two alternate methods may be used for placement:

1. An elongated, horizontal perforation is located in the top left area of the graft connecting two adjacent perforations. Proper orientation can be noted when the graft is placed on the wound site such that this horizontal perforation remains visually in the top left area of the graft.
2. The clear side of the inner pouch is indicative of the UP side of the graft orientation. Proper placement can be achieved by removing the graft from the pouch with the clear side up and moving to the wound site with that same orientation.

Absorbable, non-absorbable suture material and/or tissue adhesives can be used to fixate EpiFix to the wound site, if desired.

Primary Dressing

- EpiFix should be covered with a non-adherent contact layer.
- EpiFix should NOT be disturbed, if possible, for several days or before the next application, if needed.
- If an infection occurs at the graft site, treat infection per institution's protocol.

Secondary Dressing

- EpiFix requires a moist wound environment. Use appropriate moisture management dressings for the wound type and treatment ideology.

Support Therapies

- EpiFix is compatible with offloading/compression/negative pressure therapies.
- EpiFix can be used in conjunction with hyperbaric oxygen therapy.

Re-application of EpiFix

- It is recommended that EpiFix grafts are applied weekly until wound epithelialization is achieved. However, clinician discretion should be used based on patient and wound condition/progress. It is clinically acceptable to apply EpiFix on a biweekly basis if desired.

Adverse Effects & Reporting

- As with any procedure, the possibility of infection exists.
- Proprietary processing and validated sterilization methods are employed to eliminate potential deleterious components of the allograft. However, as with all biological implants, the possibility of rejection exists.
- Any adverse reactions, including the suspected transmission of disease attributable to this allograft, should be reported immediately to MiMedx®.

Acceptable Storage

EpiFix allografts should be stored in a clean, dry environment at ambient conditions. EpiFix allografts have a 5 year shelf life. Check the label for the expiration date.

Recovery & Quality Control

All tissue recovered meets stringent specifications during donor screening and laboratory testing to reduce the risk of transmitting infectious disease. EpiFix allografts are procured and processed in the United States according to standards and/or regulations established by the American Association of Tissue Banks (AATB) and the United States Food & Drug Administration (FDA). All tissues are recovered under full informed consent of the donors (mothers of the newborn children). The donors have consented to the transfer of the allografts to third parties. A thorough medical and social history of the donor is also obtained.

Recovery & Quality Control (cont.)

The listed communicable disease testing was performed by a laboratory registered with FDA to perform donor testing and certified to perform such testing on human specimens in accordance with the Clinical Laboratory Improvement Amendments of 1988 (CLIA) and 42 CFR part 493, or that has met equivalent requirements as determined by the Centers for Medicare and Medicaid Services (CMS).

The donor is screened for:

HIV-1&2 Plus 0 Antibody	Hepatitis B Surface Antigen
HIV Type 1 (Nucleic Acid Test (NAT))	Hepatitis C Antibody
HTLV-1&2 Antibody	Hepatitis C Virus (Nucleic Acid Test (NAT))
Syphilis (Serologic Test)	Hepatitis B Virus (Nucleic Acid Test (NAT))
Hepatitis B Core Antibody	West Nile Virus (Nucleic Acid Test (NAT))*

**WNV NAT screening conducted on donors recovered beginning February 1, 2017.*

All tests produced negative results and were reviewed prior to the release of the tissue. Only tissue from donors with acceptable test results, according to the standards of MiMedx Tissue Services, LLC, as well as the standards and/or regulations of all state and federal regulatory bodies, are released.

The infectious disease test results, consent documents, donor medical history, behavior risk assessment according to current public health services guidelines, physical assessment, available relevant medical records, as well as information from other sources or records that may pertain to donor suitability, and tissue procurement test results, have been evaluated by the MiMedx Medical Director and are sufficient to indicate that the donor suitability criteria current at the time of tissue recovery have been met.

The names and addresses of the testing laboratories, the listing and interpretation of all required infectious disease tests, a listing of the documents reviewed as part of the relevant medical records, and the name of the person or establishment determining the suitability of this allograft are on file and available upon request.

Donated Human Tissue. This allograft has been determined to be suitable for transplantation.

Allograft Processing/Preservation/Sterilization

EpiFix allografts are processed based upon strict, quality-controlled protocols that have demonstrated bioburden control. An additional assurance of safety is achieved by terminally sterilizing each allograft. Based upon validations, each graft has been effectively sterilized using e-beam irradiation. The allografts are processed with aminoglycoside antibiotics.

Recipient Tracking

The FDA requires that recipient records be maintained for the purpose of tracking the allograft following transplantation. The authorized medical professional must complete the enclosed Tissue Utilization Record, attach a peel-off, allograft-tracking label provided, and mail to the distributor (postage-paid). Please use the remaining peel-off, allograft-tracking labels for patient and hospital records.

Caution: This product must be administered by an authorized medical professional.

The user shall be solely responsible for determining the adequacy and appropriateness of the allograft for any and all uses to which the user shall apply the allograft.

MiMedx's dehydrated Human Amnion/Chorion Membrane (dHACM) allografts are now described in an official U.S. Pharmacopeia – National Formulary monograph with the publication of USP 40 - NF 35.

USP U.S. Pharmacopeial Convention

Processed with:

purion® process

Processing and donor suitability performed by MiMedx Tissue Services, LLC
Patents and patents pending see: www.mimedx.com/patents
EpiFix®, PURION®, and MiMedx® are registered trademarks of MiMedx Group, Inc. 1775 West Oak Commons Court NE, Marietta, GA 30062

©2017 MiMedx Group, Inc.
All Rights Reserved. **mimedx.com**

ES101.004
