# Supplementary material for: Mohs Defect Repair with Dehydrated Human Amnion/Chorion Membrane
Source: Facial Plast Surg Aesthet Med. 2022 Jan 3;24(1):48–53. doi: 10.1089/fpsam.2021.0167 (PMC8783622; doi:10.1089/fpsam.2021.0167)
Supplement: Supplemental data [file Suppl_FigureS1-S3.docx]

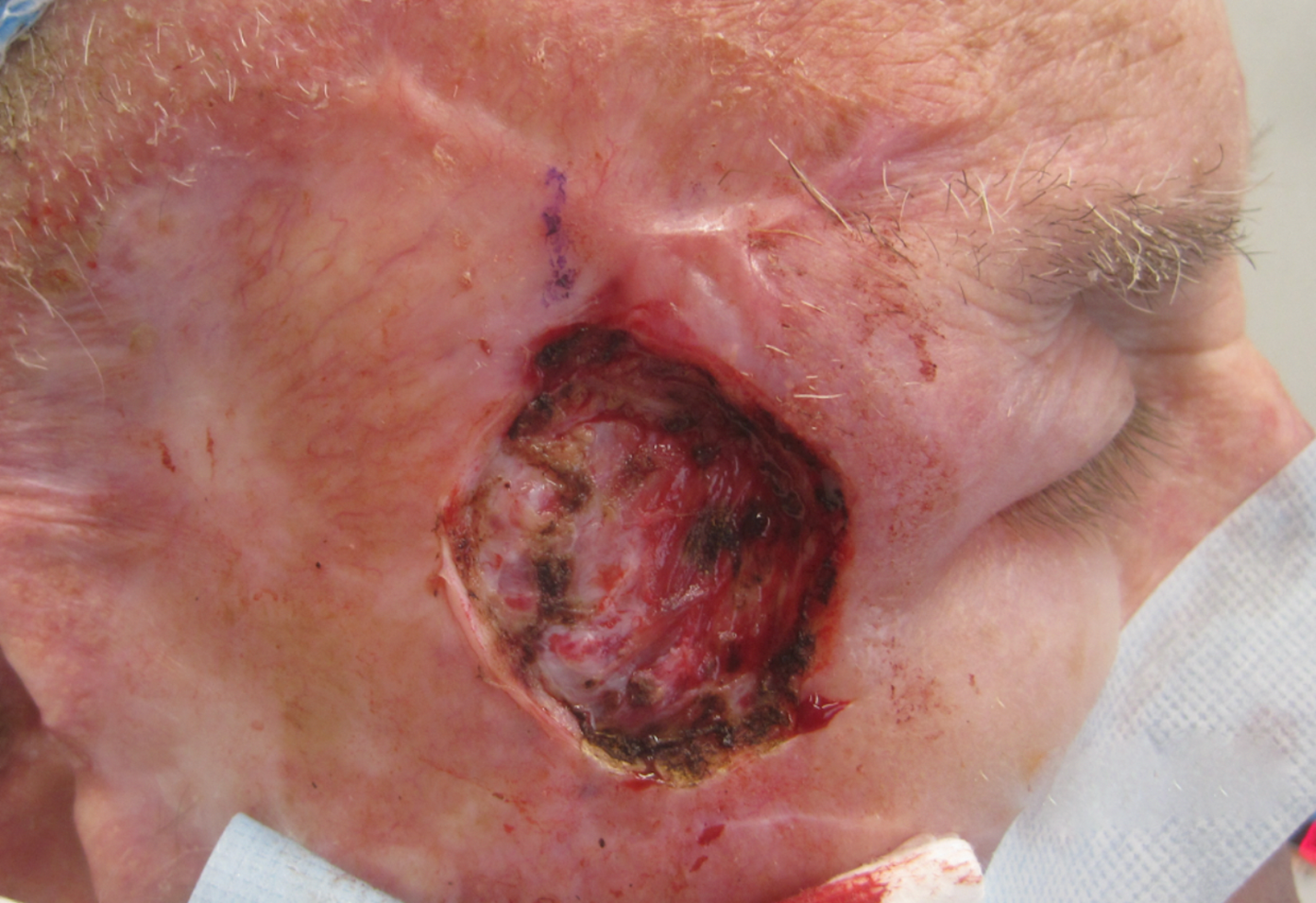

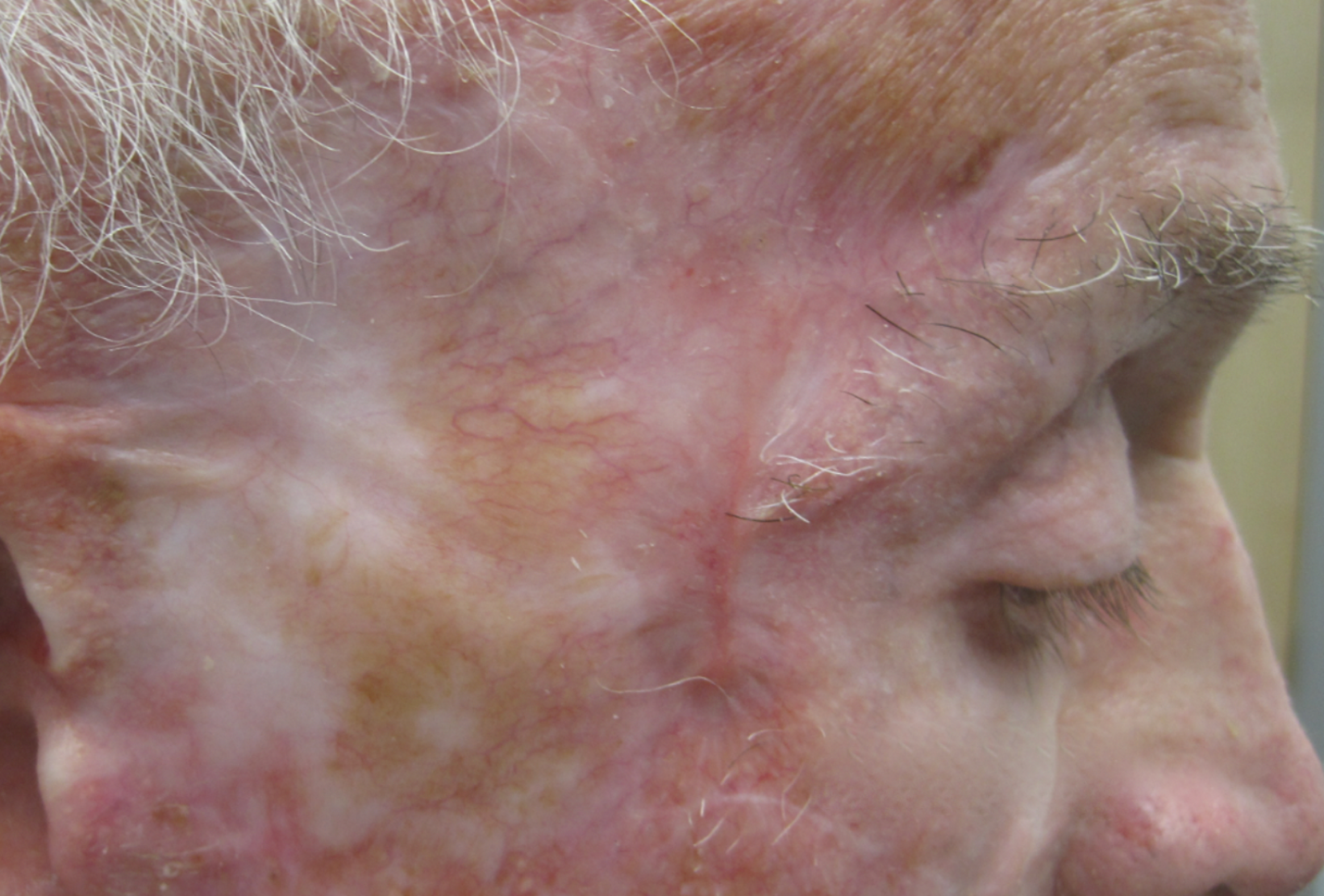


Supplemental Figure 1. Placental Allograft – Case Example

**a.** Full-thickness Mohs defect of the right lateral temple with preexisting scarring and atrophy of surrounding tissues. **b.** Result 52 days after using allograft for repair, without distortion of nearby eyelids or other critical structure.


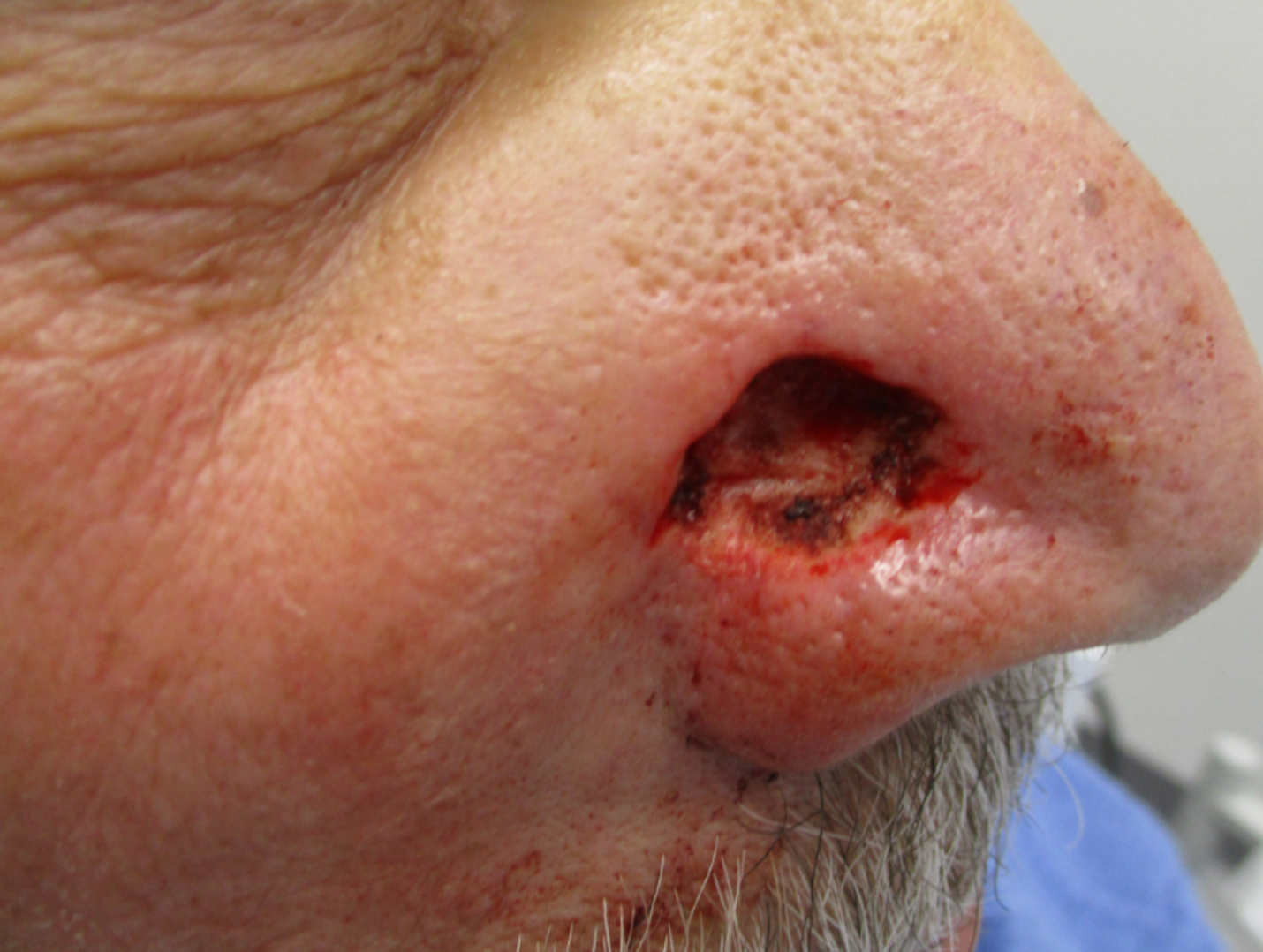

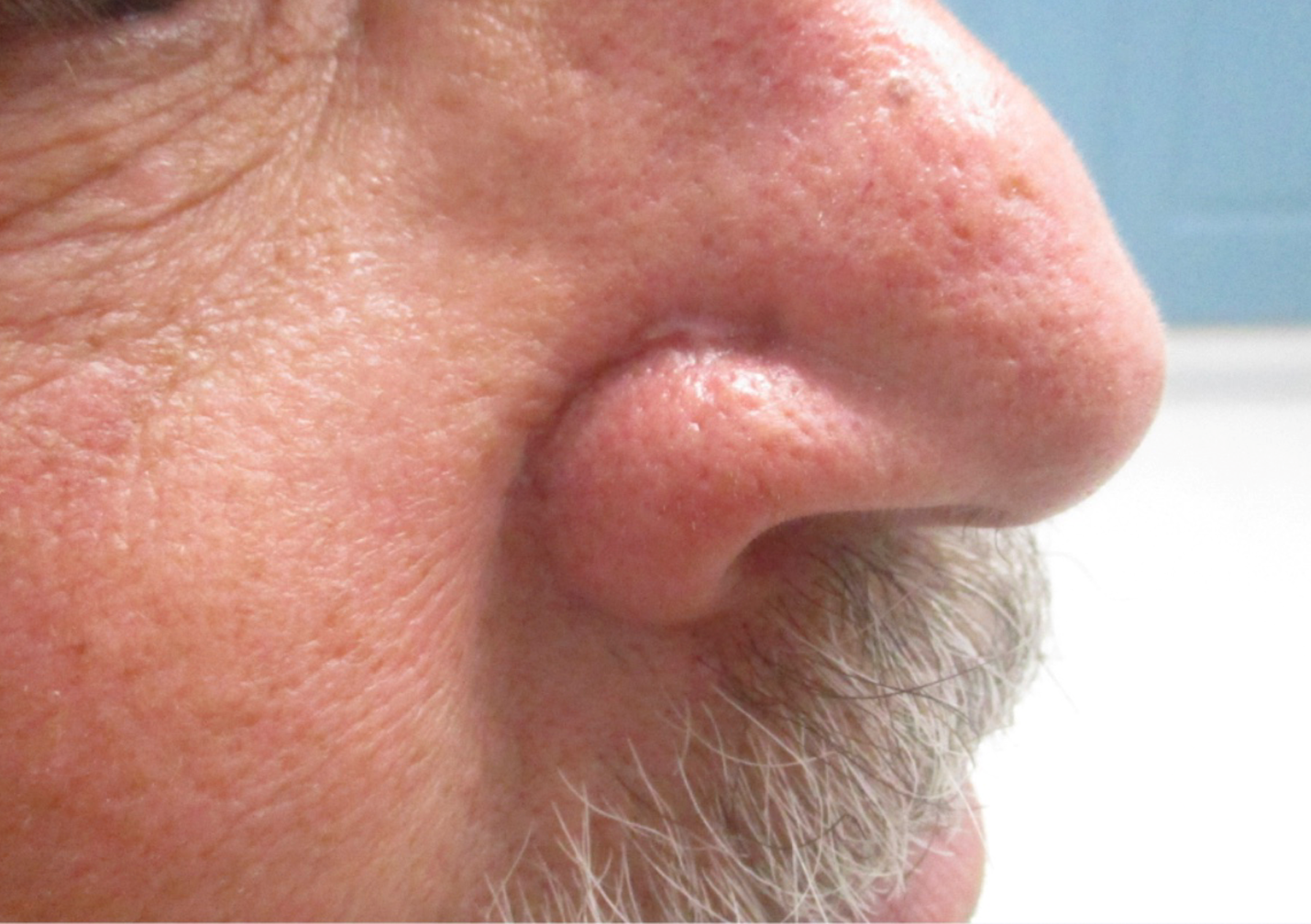


Supplemental Figure 2. Placental Allograft – Case Example

**a.** Full-thickness Mohs defect of the right nasal ala and supra-alar crease, patient refused incisional flap repair. b. Result 30 days after using allograft for repair, without contraction or distortion of surrounding tissues.


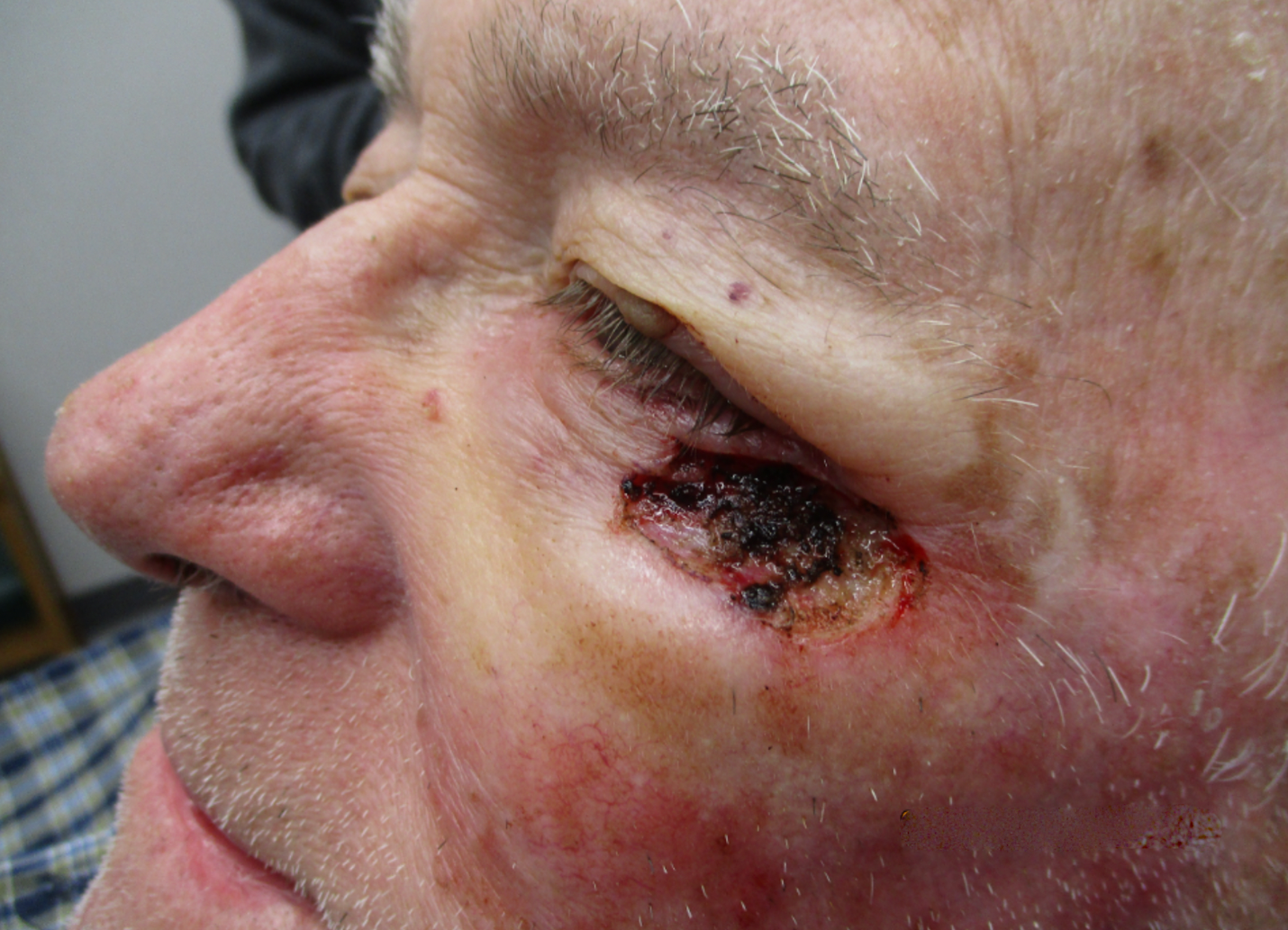

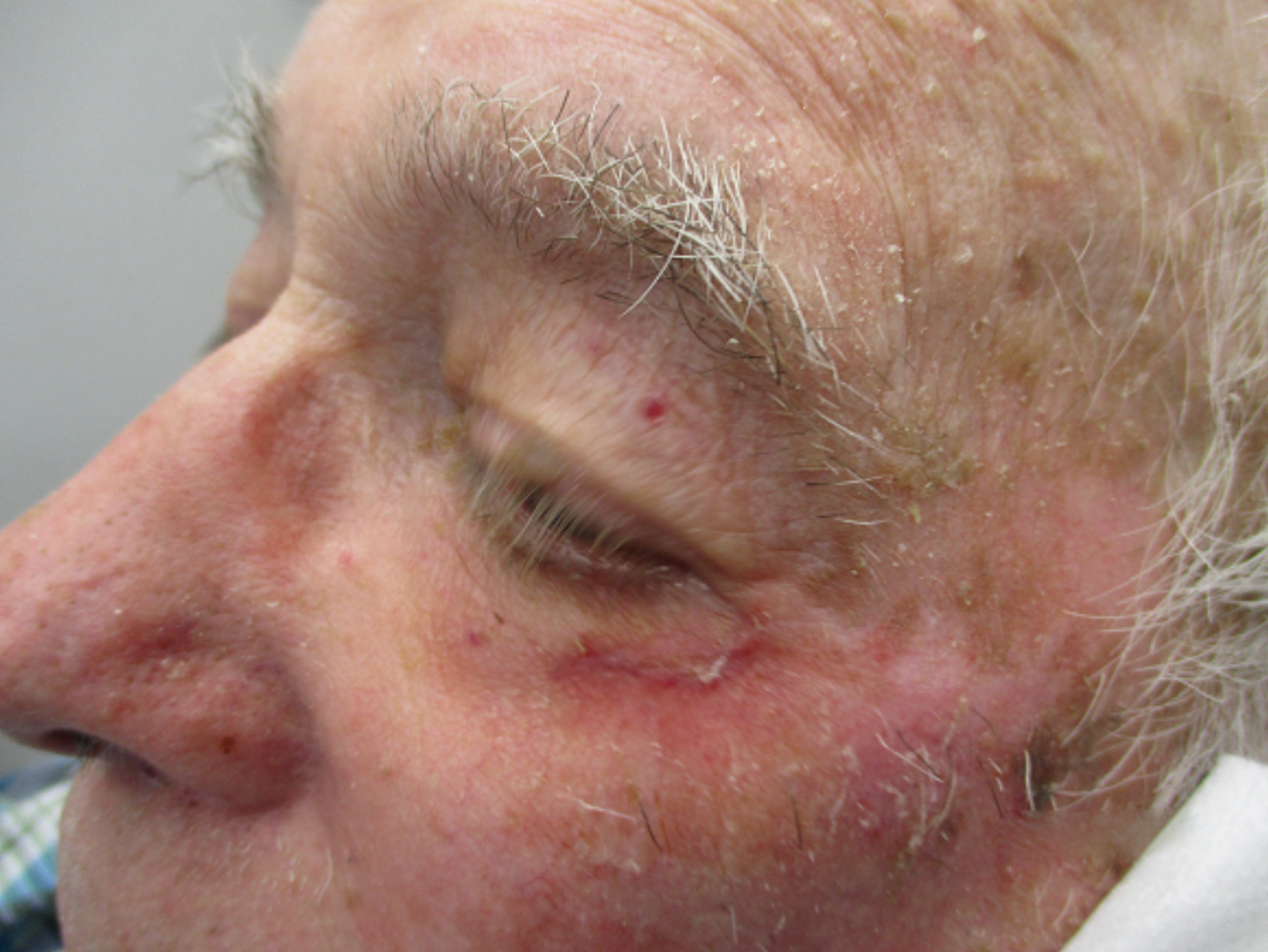


Supplemental Figure 3. Placental Allograft – Case Example

**a.** Full-thickness Mohs defect of the left lower eyelid at the lid-cheek junction. **b.** Result 36 days after using allograft for repair, without contraction or distortion of lower eyelid.
