# Supplementary material for: Mohs Defect Repair with Dehydrated Human Amnion/Chorion Membrane
Source: Facial Plast Surg Aesthet Med. 2022 Jan 3;24(1):48–53. doi: 10.1089/fpsam.2021.0167 (PMC8783622; doi:10.1089/fpsam.2021.0167)
Supplement: Supplemental data [file Suppl_FigureS4.docx]

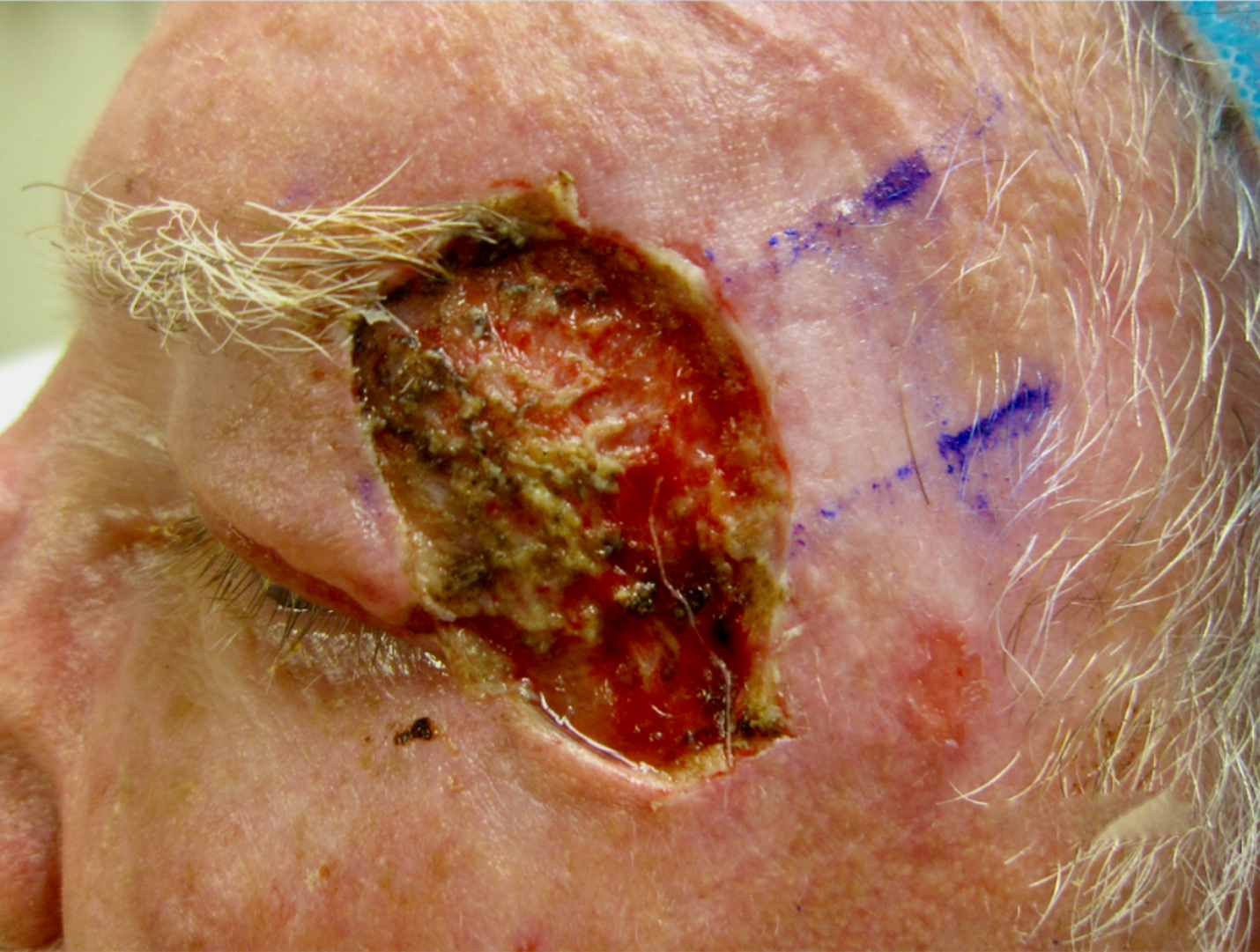

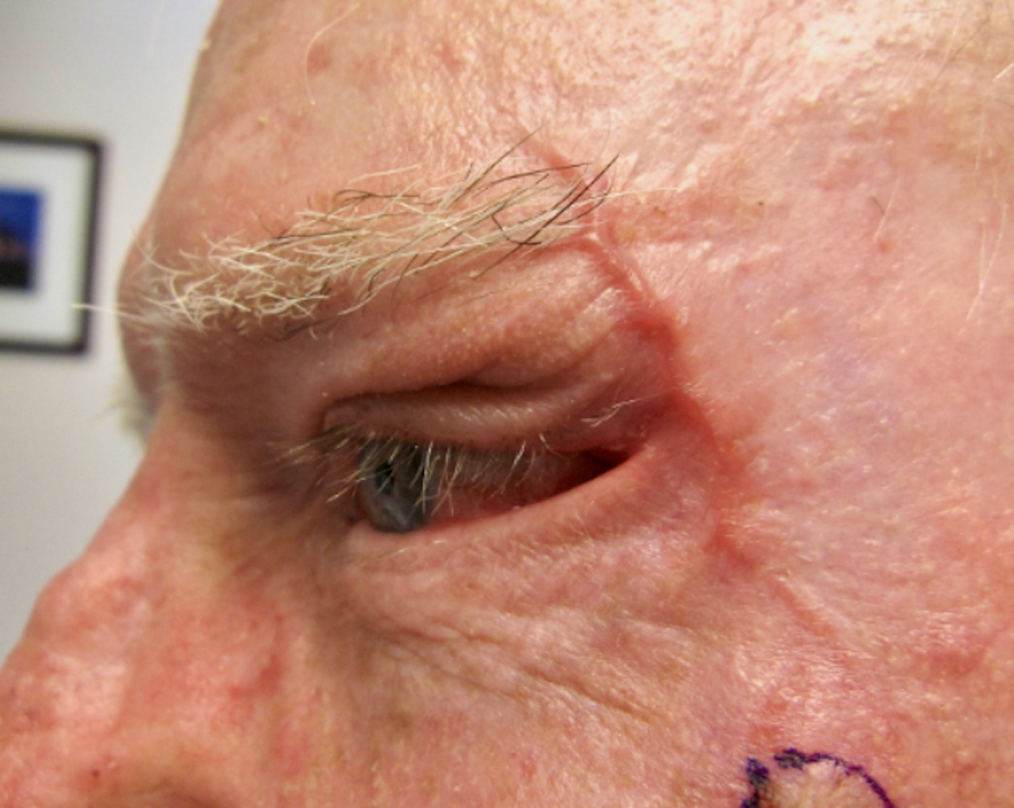


Supplemental Figure 4. Autologous Tissue – Case Example

**a.** Full-thickness Mohs defect of left lateral brow and temple. b. Postoperative result at 40 days after a FTSG closure which resulted in deformity of the lateral canthus and eyelids.
