# Supplementary material for: Mohs Defect Repair with Dehydrated Human Amnion/Chorion Membrane
Source: Facial Plast Surg Aesthet Med. 2022 Jan 3;24(1):48–53. doi: 10.1089/fpsam.2021.0167 (PMC8783622; doi:10.1089/fpsam.2021.0167)
Supplement: Supplemental data [file Suppl_FigureS5.docx]

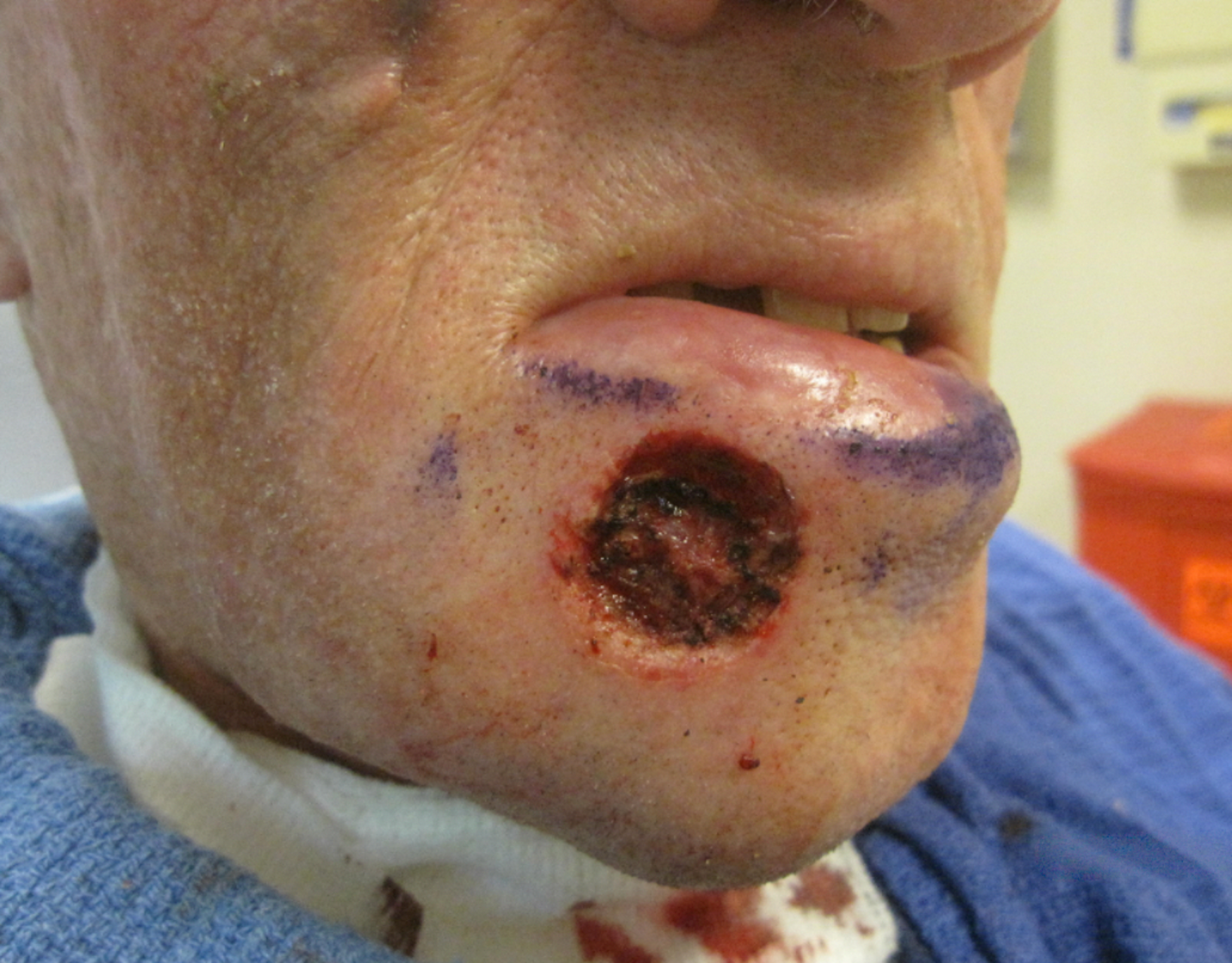


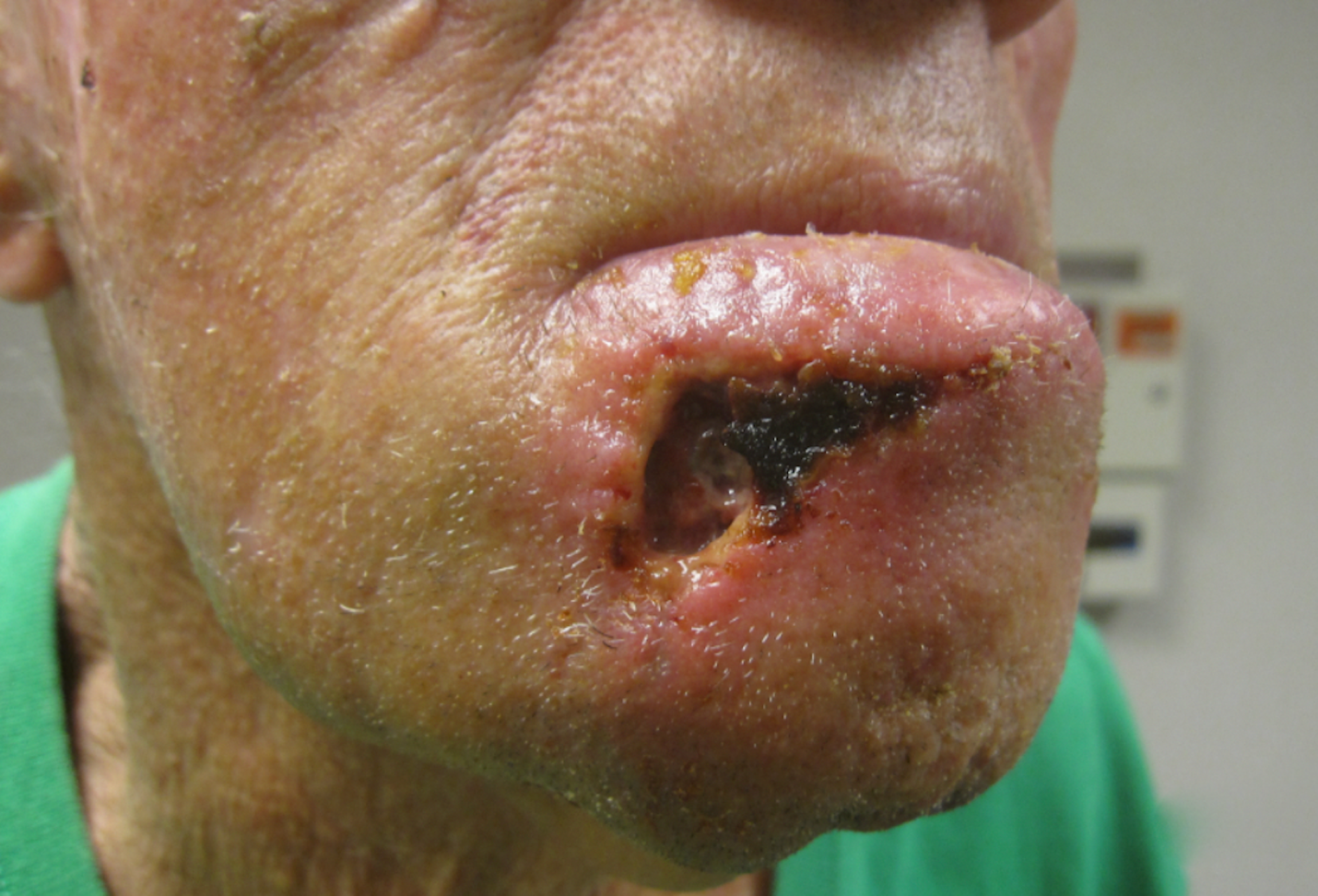


Supplemental Figure 5. Autologous Tissue – Case Example

**a.** Full-thickness Mohs defect of right inferior lip. **b.** Initial result after 12 days following O-T flap closure and subsequent infection and dehiscence.
